# Acute kidney injury may impede results after transcatheter aortic valve implantation

**DOI:** 10.1093/ckj/sfaa179

**Published:** 2020-11-03

**Authors:** Anja Haase-Fielitz, Fiona Altendeitering, Ragna Iwers, Veronika Sliziuk, Sophie Barabasch, Marwin Bannehr, Valentin Hähnel, Michael Neuss, Michael Haase, Christian Apfelbacher, Christian Butter

**Affiliations:** 1 Department of Cardiology, Heart Center Brandenburg Bernau & Faculty of Health Sciences Brandenburg, Brandenburg Medical School (MHB), Neuruppin, Germany; 2 Institute of Social Medicine and Health Economics, Otto von Guericke University Magdeburg, Magdeburg, Germany; 3 MVZ Diaverum, Diaverum, Germany; 4 Medical Faculty, Otto von Guericke University Magdeburg, Magdeburg, Germany

**Keywords:** acute kidney injury, incidence, renal complication, risk factors, renal non-recovery, TAVI

## Abstract

**Introduction:**

Severe complications after transcatheter aortic valve implantation (TAVI) are rare due to increasing procedural safety. However, TAVI procedure-related haemodynamic instability and increased risk of infection may affect renal functional reserve with subsequent renal acidosis and hyperkalaemia.

**Objective:**

In this study, we investigated incidence, modifiable risk factors and prognosis of acute kidney injury (AKI) and AKI complicated by hyperkalaemia, pulmonary oedema or metabolic acidosis after TAVI.

**Methods:**

In a retrospective single-centre study, 804 consecutive patients hospitalized during 2017 and 2018 for elective TAVI were included. AKI was defined according to the *‘*Kidney Disease Improving Global Outcome’ (KDIGO) initiative. Variables on co-morbidities, intra-/post-interventional complications and course of renal function up to 6 months after index-hospitalization were assessed. In multivariate regression analyses, risk factors for the development of AKI, complicated AKI, renal non-recovery from AKI and in-hospital mortality were determined.

**Results:**

Incidence of AKI was 13.8% (111/804); in-hospital mortality after TAVI was 2.3%. AKI was an independent risk factor for in-hospital mortality, odds ratio (OR) 10.3 (3.4–31.6), P < 0.001, further increasing to OR = 21.8 (6.6–71.5), P < 0.001 in patients with AKI complicated by hyperkalaemia, pulmonary oedema or metabolic acidosis, *n* = 57/111 (51.4%). Potentially modifiable, interventional factors independently associated with complicated AKI were infection [OR = 3.20 (1.61–6.33), P = 0.001] and red blood cell transfusion [OR = 5.04 (2.67–9.52), P < 0.001]. Valve type and size, contrast volume and other intra-interventional characteristics, such as the need for tachycardial pacing, did not influence the development of AKI. Eleven of 111 (9.9%) patients did not recover from AKI, mostly affecting patients with cardiac decompensation. In 18/111 (16.2%) patients, information concerning AKI was provided in discharge letter. Within 6 months after TAVI, higher proportion of patients with AKI showed progression of pre-existing chronic kidney disease compared with patients without AKI [14/29, 48.3% versus 54/187, 28.9%, OR = 2.3 (95% confidence interval 1.0–5.1), P = 0.036].

**Conclusions:**

AKI is common and may impede patient outcome after TAVI with acute complications such as hyperkalaemia or metabolic acidosis and adverse renal function until 6 months after intervention. Our study findings may contribute to refinement of allocation of appropriate level of care in and out of hospital after TAVI.

## INTRODUCTION

Transcatheter aortic valve implantation (TAVI) has been established as an alternative treatment to surgical aortic valve replacement. Compared with surgical valve replacement, TAVI may reduce the risk of atrial fibrillation, bleeding and acute kidney injury (AKI) [1]. However, AKI has by far not yet been abandoned in patients undergoing TAVI. The incidence of AKI after TAVI was reported to range between 6% [2] and 41% [3]. The injured kidney is frequently involved in distant organ injury such as pulmonary oedema and arrhythmias [4] potentially diminishing favourable outcomes in patients undergoing TAVI. Several studies reported AKI to be independently associated with length of hospital stay and mortality. This may be related to TAVI acutely reducing renal functional reserve through inflammation and microemboli causing haemodynamic instability and organ dysfunction. Several risk factors of AKI after TAVI have been identified including diabetes mellitus and chronic kidney disease. A summary of the literature regarding incidence and risk factors of AKI after TAVI is shown in Supplementary data, Table S1. Of those, only a few studies focused on potentially modifiable AKI risk factors [5, 6]. Furthermore, not much information is available regarding incidence and modifiers of recovery from AKI after TAVI. To what extent AKI-related complications may play a role for patient outcome and whether or not information regarding AKI after TAVI was transmitted to the outpatients has not been described, yet.

**Table 1.** Baseline characteristics and co-morbidities in patients with and without AKI

Patient baseline characteristics	AKI, *n* = 111/804	No AKI, *n* = 693/804	P-value
Male	65/111 (58.6 %)	324/693 (46.8 %)	0.021
Age, years	83 (77–86)	81 (78–85)	0.26
Body mass index, kg/m^2^	27.1 (25.3–31.1)	27.2 (24.1–30.5)	0.36
Co-morbidities, *n* (%)			
Arterial hypertension	97 (87.4)	521 (75.2)	0.008
Coronary heart disease	84 (75.7)	455 (65.7)	0.037
Chronic kidney disease	86 (77.5)	353 (50.9)	<0.001
Diabetes mellitus	51 (45.9)	273 (39.4)	0.191
Percutaneous coronary intervention	46 (41.4)	296 (42.7)	0.801
Malignant disease	23 (20.7)	174 (25.1)	0.318
Chronic obstructive disease	15 (13.5)	110 (15.9)	0.524
Coronary artery bypass grafting	19 (17.1)	66 (9.5)	0.016
NYHA III	78 (70.3)	475 (68.5)	0.715
NYHA IV	7 (6.6)	35 (5.1)	0.581
Ejection fraction in %	52.5 (40.0–60.0)	55.0 (45.0–60.0)	0.078
Logistic EuroSCORE in %	15.3 (9.0–21.9)	12.1 (8.4–19.8)	0.129

NYHA, New York Heart Association.

The objective of the present study was to identify potentially modifiable, independent, procedural risk factors for TAVI-associated AKI, to focus on renal recovery and long-term renal function. Also, the study assessed cross-sectoral information transmission regarding AKI after TAVI.

## MATERIALS AND METHODS

### Patients

**FIGURE 1:**
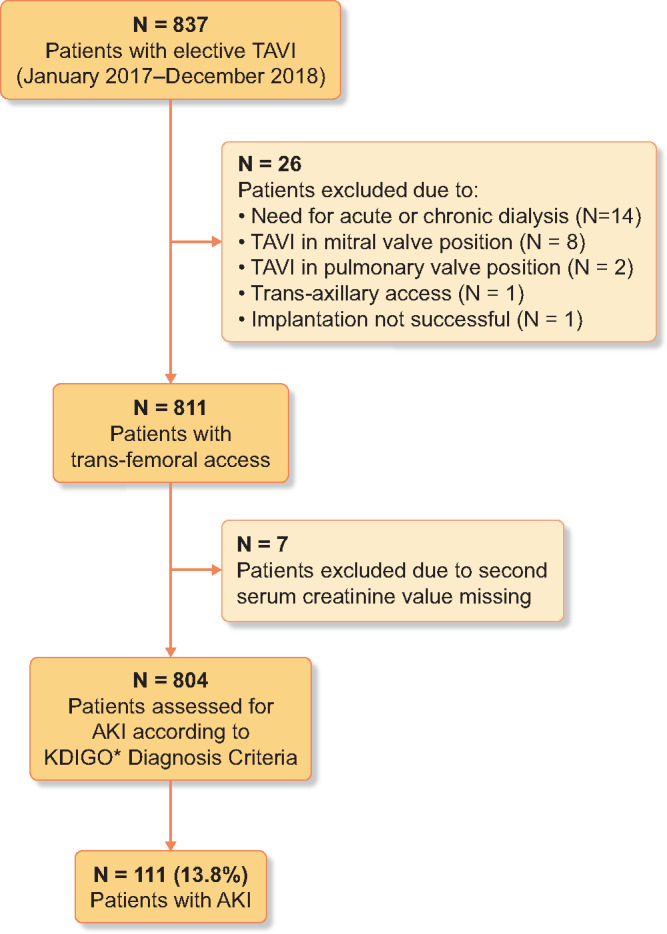
Patient flow through the study. KDIGO [8].

In this single-centre, retrospective cohort study, we analysed data from *n* = 804 consecutive adult patients (age >18 years) who were admitted to Heart Center Brandenburg in Bernau of the Brandenburg Medical School for elective TAVI procedure from January 2017 until December 2018. Patients with symptomatic severe aortic stenosis were offered TAVI if they were considered to have high-operative risk according to guidelines of the European Society of Cardiology [7]. An interdisciplinary heart valve team including interventional cardiologists and cardiothoracic surgeons was involved in allocation of surgical or non-surgical treatment of all patients. Patients were excluded if they were on acute or chronic dialysis or a second serum creatinine value within index hospital admission was missing. Patient flow through the study is shown in Figure 1. The Ethical Committee of Brandenburg Medical School approved this study and waived patient written informed consent (E-01-20190322).

### TAVI procedure

The majority of patients were admitted on the day before the procedure. TAVI was performed in the hybrid catheterization laboratory under fluoroscopy guidance with the use of contrast media. Contrast media was administered to confirm correct positioning of catheters and valve as well as final assessment of possible residual perivalvular aortic regurgitation. Patients with estimated glomerular filtration rate (eGFR) <60 mL/min received saline intravenous (IV) fluids before and after receiving contrast media when undergoing TAVI. If patients had an indication for acetylsalicylic acid, that is, coronary heart disease or peripheral vascular disease, it was continued throughout the hospital stay. Since the cohort of patients undergoing TAVI is elderly with numerous co-morbidities, a large number received angiotensin-converting enzyme inhibitors, angiotensin 2 receptor 1 (AT2) blockers, aldosterone antagonists and loop diuretics, especially when left ventricular function was reduced. These agents were withheld on the day of TAVI.

The TAVI device was delivered through femoral approach in all patients. Either self-expandable or balloon-expandable valve prosthesis was used. Procedures were performed under local anaesthesia with conscious sedation or general anaesthesia with endotracheal intubation. The prosthesis size was determined using pre-procedural echocardiographic and multi-slice computed tomography angiogram findings. Full blood count and renal function were obtained at admission (baseline), daily until Day 3 post-interventional and, thereafter, according to clinical need until patient discharge. After hospital discharge, patients were recommended to visit TAVI-dedicated outpatient clinic at Month 3 after TAVI, then on a yearly basis.

### Study endpoints and data collection

AKI was defined according to the Kidney Disease Improving Global Outcome (KDIGO) criteria [8]. AKI-related renal complications included intra-/post-procedural hyperkalaemia (*K* > 5.5 mmol/L), pulmonary oedema (symptoms and X-ray) and metabolic acidosis [pH <7.37, base excess (BE) <−3 and lactate <2 mmol/L]. We defined non-recovery from AKI as eGFR calculated by the Chronic Kidney Disease Epidemiology Collaboration (CKD-EPI) equation [9] not returning to ≥60% of baseline corresponding with the definition of acute kidney disease proposed by the KDIGO initiative [8].

Medical records and databases were reviewed until hospital discharge and the following information was obtained: demographics, co-morbidities, procedural characteristics including type and size of valve, complications during the hospital stay (intra-/post-procedural: cardiac decompensation, need for packed red blood cells, delirium, need for *de novo* pacemaker implantation), laboratory parameters (serum creatinine, pH, potassium and base excess) and length of stay in hospital after TAVI, 30-day rehospitalization and in-hospital mortality. Infection was defined as note in electronic medical record.

In a subgroup of patients, follow-up serum creatinine concentration was available within 6 months after index hospitalization due to presentation at outpatient clinic or due to rehospitalization.

Status of pre-existing chronic kidney disease was determined from baseline eGFR calculated using the CKD-EPI equation [9]. CKD progression was defined as change in CKD from Stage 3a to 3b or worse [10]. Cross-sectoral transmission of AKI-related information was assessed in patient discharge letter.

### Statistical analysis

Categorical variables were analysed with Fisher’s exact or Chi-squared test and continuous variables using non-parametric Mann–Whitney U-test. Logistic regression analysis was performed to establish risk factors for AKI, AKI with complications, renal non-recovery or in-hospital mortality after TAVI. The following variables were included in the multivariate model for prediction of AKI or AKI with complications or renal non-recovery: gender, arterial hypertension, chronic kidney disease, coronary heart disease, coronary artery bypass grafting, infection, blood transfusion (packed red blood cells), need for pacemaker implantation and cardiac decompensation. The following variables were included in the multivariate model for in-hospital mortality prediction: gender, infection, packed red blood cells and AKI with and without complications. A two-sided P-value of <0.05 was considered statistically significant. SPSS 25 (IBM, Armonk, NY, USA) was used.

## RESULTS

AKI occurred in 111/804 (13.8%) patients undergoing TAVI at our centre with five patients receiving acute renal replacement therapy. Median occurrence of AKI was 3 days post-procedure (25th–75th percentile 2–4). Patients with AKI more frequently presented with cardiovascular co-morbidities compared with patients without AKI (Table 1).

AKI was associated with increased length of stay in hospital compared with patients without AKI [8 days (25th–75th percentile 6–11) versus 6 days (5–8), P < 0.001].

In-hospital mortality after TAVI was 2.3%, being 9% for patients with AKI versus 0.7% for patients without AKI, P < 0.001 (Table 2). In multivariate analysis adjusting for gender and non-kidney-related procedural complications, AKI independently predicted in-hospital mortality with odds ratio (OR) 10.3 [95% confidence interval (CI) 3.4–31.6], P < 0.001 (Table 3).

**Table 2.** Outcome parameter in patients with and without AKI

Outcome parameter	AKI, *n* = 111/804, *n* (%)	No AKI, *n* = 693/804, *n* (%)	OR (95% CI)	P-value
In-hospital mortality	10 (9)	5 (0.7)	13.6 (4.6–40.7)	<0.001
Cardiac rehabilitation	40 (36)	386 (55.7)	0.45 (0.30–0.68)	<0.001
Geriatric rehabilitation	27 (24.3)	125 (18)	1.7 (1.04–2.7)	0.041
Rehospitalization within 30 days after index hospitalization	6 (5.4)	19 (2.7)	2.0 (0.79–5.19)	0.317

**Table 3.** Logistic regression analysis for in-hospital mortality

Risk factors	P-value univariate	P-value multivariate	OR (95% CI)
Blood transfusion post TAVI	0.014	0.004	4.9 (1.7–14.5)
Female gender	0.067	0.13	0.4 (0.1–1.3)
Infection post TAVI	0.055	0.42	1.7 (0.5-6.1)
AKI			
Overall	<0.001	<0.001	10.3 (3.4–31.6)
AKI with complications^a^	<0.001	<0.001	21.8 (6.6–71.5)

a Hyperkalaemia, pulmonary oedema and metabolic acidosis.

**FIGURE 2:**
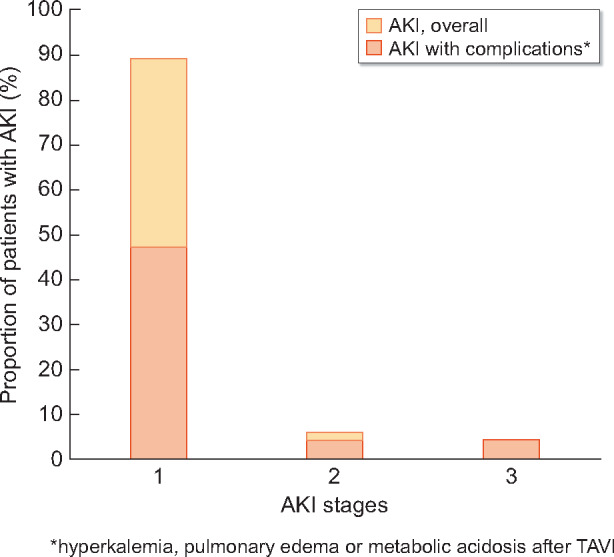
Incidence of AKI according to Stages 1–3.

In about half of patients with AKI (57/111, 51.4%), hyperkalaemia, pulmonary oedema or metabolic acidosis were observed. In AKI Stage 1, complications were found in 47.5% of patients, Stage 2: 71.4% and Stage 3: 100% of patients (Figure 2). In patients with complicated AKI, risk of hospital death increased to 21.8-fold (6.6–71.5), P < 0.001 (Table 3).

### Risk factors for AKI and AKI with complications

**FIGURE 3:**
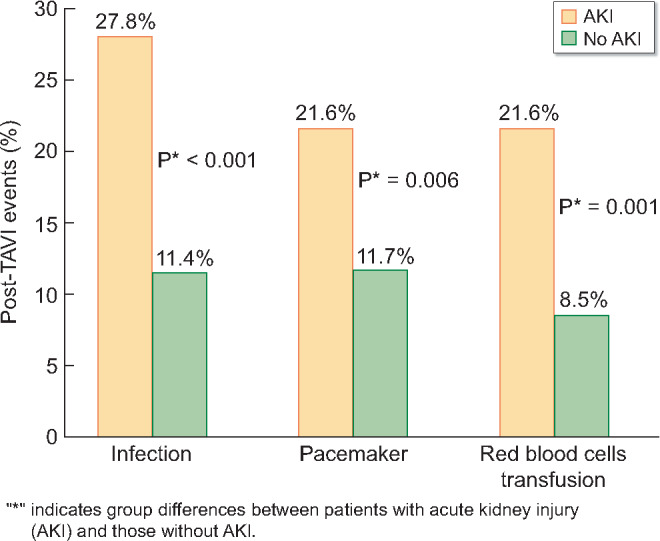
Potentially modifiable, independent peri-interventional risk factors for AKI.

Potentially ‘modifiable’, independent interventional risk factors for AKI included infection [OR = 2.52 (95% CI 1.42–4.48), P = 0.002], need for pacemaker implantation [OR = 2.01 (95% CI 1.17–3.46), P = 0.011] and red blood cell transfusion [OR = 2.51 (95% CI 1.44–4.40), P = 0.001] with unadjusted proportions shown in Figure 3. Other potentially modifiable, independent risk factors contributing to AKI such as type and size of implanted valve, need for tachycardial pacing, pre-interventional use of renn angiotensin, aldosteron system (RAAS) inhibitors and volume of contrast agent used during the intervention were not independently associated with AKI (Table 4). In multivariate regression analysis, ‘non-modifiable’ risk factors for AKI were chronic kidney disease [OR = 2.95 (95% CI 1.82–4.78), P < 0.001], arterial hypertension [OR = 2.01 (95% CI 1.10–3.77), P = 0.023] and male gender [OR = 1.70 (95% CI 1.25–2.63), P = 0.014].

**Table 4.** Potentially modifiable risk factors contributing to AKI

Risk factors	AKI, *n* = 111/804, *n* (%)	No AKI, *n* = 693/804, *n* (%)	P-value
Self-expanding valve	74 (66.7)	483 (69.7)	0.521
Ballon-expanding valve	37 (33.3)	210 (30.3)	0.521
Valve size			
23 mm	17 (15.3)	108 (15.6)	0.863
25 mm	4 (3.6)	15 (2.2)	
26 mm	24 (21.6)	169 (24.4)	
27 mm	2 (1.8)	25 (3.6)	
29 mm	42 (37.8)	250 (36.1)	
31 mm	2 (1.8)	8 (1.2)	
34 mm	20 (18)	117 (16.9)	
Type of valve, *n* (%)			
Evolut™ (Pro, *R*)	63 (56.8)	419 (60.5)	0.948
SAPIEN™ (XT and 3)	37 (33.3)	211 (30.4)	
ACURATE neo™	5 (4.5)	32 (4.6)	
NVT ALLEGRA™	6 (5.4)	30 (4.3)	
Portico™	0 (0)	1 (0.1)	
Valve-in-Valve	7 (6.3)	46 (6.6)	0.896
Tachycardia pacing	90 (81.1)	610 (88)	0.587
Pre-dilatation	79 (71.2)	513 (74)	0.460
Volume of contrast agent, mL (median (25th - 75th percentile))	125 (101–160)	126 (101–156)	0.969
RAAS inhibitors	83 (74.8)	558 (80.5)	0.162

Self-expanding valve: Evolut™, ALLEGRA™, ACURATE neo™ and Portico™; Ballon-expanding valve: SAPIEN™.

Independent risk factors for AKI with complications were chronic kidney disease [OR = 3.02 (95% CI 1.54–5.94), P = 0.001], male gender [OR = 2.33 (95% CI1.28–4.17), P = 0.005], infection [OR = 3.20 (95% CI 1.61–6.33), P = 0.001] and red blood cell transfusion [OR = 5.04 (95% CI 2.67–9.52), P < 0.001].

### AKI-related complications and non-recovery from AKI

AKI was associated with higher proportion of patients developing delirium (9%) compared with patients without AKI (4%), OR = 2.4 (1.1–5.0), P = 0.022.

Eleven of 111 (9.9%) patients did not recover from AKI. Patients with cardiac decompensation less frequently recovered from AKI (5/11, 45.5%) compared with patients with renal recovery (12/100, 12%), P = 0.003. Independent risk factors for renal non-recovery from AKI were cardiac decompensation [OR = 3.03 (95% CI 1.39–6.67), P = 0.005], infection [OR = 2.56 (95% CI 1.15–5.88), P = 0.021] and the need for blood transfusion [OR = 4.35 (95% CI 2.04–9.09), P < 0.001]. Co-morbidities such as diabetes or chronic kidney disease, medication or procedural characteristics were not predictors of renal non-recovery from AKI after TAVI. In patients without renal recovery, length of stay in intensive care was prolonged [3 days (25th–75th percentile 0–8)] compared with patients with renal recovery [0 days (0–0), P < 0.001] and in-hospital mortality was increased (63.6% versus 3%, P < 0.001). Four of 11 patients with renal-non-recovery survived index hospital stay. Out of them, two patients are still alive 2 years after TAVI. Both are not on chronic dialysis.

### Course of renal function after TAVI

Information on severity or cause of AKI in discharge letter was provided in 18/111 (16.2%) patients. In 217/804 (27.0%) patients, renal function was measured within 6 months after AKI. Within 6 months after TAVI, higher proportion of patients with AKI showed progression of pre-existing chronic kidney disease compared with patients without AKI [14/29, 48.3% versus 54/187, 28.9%, OR = 2.3 (95% CI 1.04–5.09), P = 0.036].

## DISCUSSION

In 804 consecutive TAVI patients enrolled over 2 years, we found AKI to occur frequently and usually 3 days post-interventional. In almost half of patients with AKI, kidney-related complications occurred, even in patients with AKI Stage 1. In patients with AKI after TAVI, hospital stay was about 2 days longer compared with those without AKI. AKI was associated with >10-fold increased in-hospital mortality, even more pronounced in patients with complicated AKI. We identified potentially modifiable, interventional risk factors of complicated AKI including post-TAVI red blood cell transfusion and infection. Also, non-recovery from AKI until hospital discharge occurred in about 10% of patients frequently associated with cardiac decompensation. Within 6 months after TAVI, a higher proportion of patients with AKI during index-hospital stay showed progression of pre-existing chronic kidney disease compared with patients without AKI. Finally, cross-sectoral transmission of AKI-related information in thepatient discharge letter was provided in every sixth affected patient.

Previous studies reported AKI occurring even in populations without pre-existing chronic kidney disease [11]. AKI incidence after TAVI appears to range between 11% [12] and 41% [3], usually with >80% of patients exhibiting AKI Stage 1 [3, 13]. Independent AKI predictors included bleeding and infection after TAVI [13, 14]. Further research is needed on how to avoid infection and bleeding for kidney protection.

Non-recovery from AKI was shown to occur in 13% of affected patients [15]. Causes for AKI non-recovery comprised the presence of chronic kidney disease, diabetes mellitus and blood transfusion [15]. AKI was demonstrated to be an independent risk factor for in-hospital mortality [5, 16, 17].

None of studies reported on timing of AKI occurrence in relation to TAVI procedure or AKI-related complications such as hyperkalaemia, metabolic acidosis or pulmonary oedema. Also, both cross-sectoral information transmission regarding AKI and long-term follow-up of kidney function have not been reported, yet.

AKI is a concern with a high incidence and clinical burden among patients across acute care settings such as increasingly performed TAVI. Rapid identification and treatment of AKI and AKI-related complications have been shown to improve patient outcomes in clinical settings other than TAVI, such as critical care [18]. Quality of care to patients after TAVI may be further improved to maintain procedure-related patient benefit. Therefore, further understanding of the impact of AKI on length of hospital stay and recognition of potential risk modifiers of acute care is needed. Understanding the timing of AKI, factors of AKI non-recovery, obstacles of cross-sectoral information transmission and course of long-term kidney function may refine allocation of the appropriate level of care in and out of hospital after TAVI. Recognition of AKI-related complications, regardless of AKI stage, may enable their effective early treatment.

Although TAVI may reduce risk of AKI compared with surgical valve replacement, AKI still affected >10% of patients in our study. This result is in line with studies on the lower end of reported AKI incidence [2, 12] and may reflect elective patient population, low volume of contrast agent applied and TAVI procedure performed in a high-volume centre. Also, in all study patients, transfemoral access was chosen. Performance of transapical TAVI access was not clinical practice in our centre during the study period. This is especially important because transapical TAVI access has been reported to be associated with higher in-hospital mortality and higher rates of AKI compared with transfemoral TAVI access [19]. Knowledge of the occurrence of AKI ∼3 days after TAVI may guide clinician awareness including prescribing kidney function tests and assessment of kidney risk factors until hospital discharge. Of note, a substantial proportion of patients developed AKI-related complications associated with >20-fold increased risk of hospital mortality even after adjustment for classical risk factors. As expected, almost all patients with AKI Stages 2 and 3 showed hyperkalaemia, metabolic acidosis or pulmonary oedema, whereas still >50% of patients with AKI Stage 1 were affected by these kidney-related complications. Accordingly, even patients with AKI Stage 1 may benefit from additional attention on renal function. Whether patients with complicated AKI exhibit adverse outcome or whether patients with uncomplicated AKI may receive downgraded clinical attention may be addressed by future studies.

The present finding of AKI prolonging hospital stay by about 2 days is similar in magnitude to what other studies reported [16, 20]. Such impaired patient outcome may call for complex preventive measures such as implementation of AKI eAlert systems, kidney care bundles and quality indicators including involvement of the outpatient sector as proven successful in settings other than TAVI [18, 21–26]. Identified kidney risk factors such as bleeding, infection, chronic kidney disease or arterial hypertension are difficult, if not impossible, to be modified for TAVI procedure.

Although most affected patients demonstrated recovery from AKI until hospital discharge, a significant proportion of patients was discharged with non-recovery of kidney function. Interestingly, kidney non-recovery was frequently associated with cardiac decompensation post-TAVI emphasizing the need for the best possible pre-procedural risk assessment and patient selection, optimized cardiac drug therapy and volume management and post-TAVI cardiorenal function monitoring. However, categorization of kidney non-recovery could not be done given the relatively low rate of this event. Finally, cross-sectoral AKI-related information transmission was provided in only a few affected patients and a considerable proportion of patients with AKI showed long-term progressing chronic kidney disease. Whereas improved cross-sectoral information transmission appears to be doable and reasonable, improvement in information transmission may lead to improved kidney outcome after TAVI when combined with other kidney-protective strategies including kidney-specific follow-up of patients after TAVI.

We were able to include a relatively high number of patients in a relatively short period of time reducing potential performance bias. Our study enabled in-depth analysis of clinical data including various procedure-related variables, analysis of acid–base and electrolyte status and follow-up of kidney function 6 months after TAVI. As far as we know, this study is the first reporting on the relevance of AKI-related complications.

Although only few studies were larger [2, 13, 20] than our study and the present study was the first to report on the lack of cross-sectoral information transmission, we were not able to clarify the potential effect of information loss on care and outcomes of patients with AKI after TAVI.

Relatively low absolute numbers of patients with non-recovery from AKI precluded categorization and robust identification of independent AKI-recovery modifiers. Also, we were not able to clarify AKI aetiology in relation to timing of AKI after TAVI. Finally, serum creatinine measurement was done according to clinical decision, and daily creatinine measurement was not available in all patients, which may have affected the incidence of AKI and non-recovery from AKI.

## CONCLUSION

AKI is common and may impede patient outcome after TAVI. Hyperkalaemia, pulmonary oedema or metabolic acidosis may occur in about half of patients with AKI. Until 6 months after AKI, progression of pre-existing chronic kidney disease is frequently observed. Our study findings may contribute to the understanding of timing of AKI, relevance of AKI complications, factors of AKI non-recovery and magnitude of lacking cross-sectoral information transmission. Therefore, our study may contribute to refinement of allocation of the appropriate level of care in and out of hospital after TAVI.

## SUPPLEMENTARY DATA

Supplementary data are available at ckj online.

## STATEMENT OF ETHICS

This study was approved by the Ethics Committee of the Brandenburg Medical School (MHB), E-01-20190322.

## AUTHORS’ CONTRIBUTIONS

A.H.-F. designed the study; F.A. obtained the data; A.H.-F., F.A., and M.H. analyzed the data; A.H.-F., F.A. and M.H. made the figures; A.H.-F. and M.H. drafted the paper; all authors revised and approved the final version of the manuscript.

## CONFLICT OF INTEREST STATEMENT

All authors have no conflicts of interest to declare.

## Supplementary Material

sfaa179_Supplementary_Data
