# Supplementary material for: Acute kidney injury may impede results after transcatheter aortic valve implantation
Source: Clin Kidney J. 2020 Nov 3;14(1):261–8. doi: 10.1093/ckj/sfaa179 (PMC7857802; doi:10.1093/ckj/sfaa179)
Supplement: sfaa179_Supplementary_Data [file sfaa179_supplementary_data.docx]

**Appendix Table 1** – Literature search for “AKI / TAVI / mortality / independent”

| **Reference** | **Studie Design** | **Patients** | **AKI Definition** | **Incidence**  **of AKI** | **Independent predictors of AKI** | **prognostic value** | **Independent predictors of postoperative mortality** | **Further results** |
| --- | --- | --- | --- | --- | --- | --- | --- | --- |
| Nunes Filho ACB  Rev Esp Cardiol (Engl Ed). 2019 | multicenter registry | N=794 consecutive patients | VARC-2- def | 18% | Age (OR 1.03), diabetes (OR 1.53), major bleeding (OR 3.3) and valve malpositioning  (OR 4.9) | LOS hospital  with AKI (19.3 vs  11.2 days) | all-cause death:  AKI (HR: 2.8)  -cardiovascular mortality: AKI (HR: 2.9) | -impact of AKI on mortality is limited to the first year after TAVI  -Impact access site on AKI |
| Azarbal A  Am J Cardiol 2019 | multicenter  registry | N= 1,502 consecutive patients  baseline CKD  (N=755) | AKI (decrease in eGFR >25%)  +  AKR (25% improvement in GFR at time of hospital discharge) | AKR 17.8% | AKR: moderate to  severe lung disease (OR 1.5), eGFR < 50 ml/min (OR 1.7- 3.3), DM (OR 0.6) baseline anemia (OR 0.5), STS risk score >6.1(OR 0.65) | 4 days longer hospital stay in patients with AKI vs AKR  Hospital mortality 18.8% vs. 1%in patients with AKI vs AKR | - | 2012-2017  3-group analysis: 3 groups: AKR (≥25% increase in GFR), unchanged GFR, and AKI-GFR (≥25% decrease in GFR). |
| Ando T  Am J Cardiol 2019 | administrative database | N=9,521 patients | NA | 17%-27% depending on emergency status | NA | NA | NA | AKI as independent risk factor for non-elective admission in TAVI |
| Azarbal A  Am J Cardiol 2018 | retrospective study | N=366 consecutive patients | AKI (decrease in eGFR >25%)  +  AKR (25% improvement in GFR 48 hrs after TAVI) | 14.8%  (AKR 13%) | AKI: Hemoglobin (per 1 mg/dl) (OR 0.78)  AKR: female (0.44), lack of chronic β-blocker utilization (0.49), CKD (1.04) | Hospital mortality 9.3% vs. 0%in patients with AKI vs AKR | - | 2012-2017  3-group analysis: 3 groups: AKR (≥25% increase in GFR), unchanged GFR, and AKI-GFR (≥25% decrease in GFR) |
| **Reference** | **Studie Design** | **Patients** | **AKI Definition** | **Incidence**  **of AKI** | **Independent predictors of AKI** | **prognostic value** | **Independent predictors of postoperative mortality** | **Further results** |
| Kumar N  Am J Cardiol 2018 | retrospective study | N=2,820 patients | NA | Transfemoral (TF)-TAVI with lower rates of AKI (14.6% vs 24.5%, p <0.001) compared with Transapical-(TA)-TAVI  TF-TAVI was associated with similar rates of AKI-D (1.3% vs 1.7%, p = 0.37) | NA | TF-TAVI was  associated with lower in-hospital mortality (3.1% vs 4.9%, p = 0.01) | NA | TF-TAVI is associated with lower rates of in-hospital mortality and AKI compared with TA-TAVI. |
| Nijenhuis VJ  Am J Cardiol. 2018 | retrospective study | N= 639 consecutive patients | AKIN | 10% | CKD (OR 46.9), pulmonary hypertension (OR 3.0) | 6 days longer hospital stay in AKI vs stable or improved kidney function  30 day mortality 45% vs. 2% in AKI vs improved kidney function | 2-year mortality rate: AKI (HR: 3.7) | 2007-2015  3 groups according to ratio of creatinine post- to pre-TAVI: improved kidney function 15%), stable kidney function (75%), and AKI (10%) |
| Attard S  Clin Nephrol 2018 | retrospective study | N=104 patients | AKIN / KDIGO | 35.9%  (1: 26.2%  2: 5.8%  3: 3.9%)  2.9% RRT | EuroSCORE-II  (OR: 1.19) and  CKD (OR: 2.74) | All-cause mortality 30 days, 6 and 24 months: 1,9% / 5,8%/ 20,2%  longer hospital stay (6 days vs. 5 days) | AKI (HR: 3.0) | Recruitment: 2010-2017  Dose of contrast media was not different (216 ml vs. 198 ml) |
| **Reference** | **Studie Design** | **Patients** | **AKI Definition** | **Incidence**  **of AKI** | **Independent predictors of AKI** | **prognostic value** | **Independent predictors of postoperative mortality** | **Further results** |
| Ferro CJ  JACC Cardiovasc Interv 2017 | UK-wide registry | N=6,464 patients | patients newly requiring dialysis after TAVR | 3.1% | Lower baseline eGFR (OR 0.95), LVEF<30% (OR 1.53), diabetes mellitus (OR 1.63); year of procedure (OR 0.89), non-trans-femoral approach (OR 2.46), conversion to open surgery (OR9,59), type of valve (OR 1.92) | In-hospital mortality: 4.9% (40.6% with dialysis post TAVR) | Dialysis requirements before (OR 2.46) and after TAVR (OR 3.54) and risk for 4-year mortality | new need for dialysis after TAVR is associated with higher mortality at 30 days, and 4 years than that of patients on dialysis before TAVR and patients not requiring dialysis. |
| Vavilis G Open Heart 2017 | Nationwide  registry | N=1,540 patients | VARC-2 | 6.1%  (1: 83%  2: 4%  3: 13%) | eGFR (OR 0.9), male gender (OR 2.7), apical access (OR 2.2) | Mortality at 1 year 10.4% | 1-year mortality:  AKI (HR 2.0), male (1.5), LVEF <30% (2.0), DM (1.5), PVD (1.5), AF (1.4) | 2008-2015 |
| Thongprayoon C J Card Surg. 2017 | retrospective study | N=386 patients | Persistent AKI (increased creatinine at discharge  (≥0.3 mg/dL or ≥50% from baseline): | pAKI: 13% | Diabetes (OR: 2.4), PCI (OR: 2.4), IABP (OR: 8.1), blood transfusion (OR: 2.2) Protective for pAKI: higher baseline eGFR (OR: 0.83 per 10-mL/min/1.73 m^2^ increase in eGFR) | In-hospital mortality: 2.9% (pAKI 7/50, 14% vs. no pAKI 4/336, 1.2%) | 2-year mortality after adjusting for STS risk score, pAKI (HR: 2.7) | 2008 - 2014 |
| Giannini F  Int J Cardiol 2016 | retrospective study | N=422 consecutive patients | VARC-2-def | 33% | Hyperglycemia (OR: 2.0), transfusion (OR: 2.3), EuroScore (OR: 1.2), BMI (OR: 1.1) | Overall hospital mortality 4.2% | - | Post-procedural hyperglycemia with a higher risk for AKI and mortality |
| **Reference** | **Studie Design** | **Patients** | **AKI Definition** | **Incidence**  **of AKI** | **Independent predictors of AKI** | **prognostic value** | **Independent predictors of postoperative mortality** | **Further results** |
| D'Onofrio A  Ann Thorac Surg 2016 | retrospective study | N=338 consecutive patients | VARC-2-def | 17.2% | - | All-cause 30-day mortality 4.4% | all-cause 2-year mortality: MI (OR 2.7), para-valvular leak (OR 2.5), AKI (OR 3.1) | 2007-2013 |
| Chatani K  J Cardiol 2015 | retrospective study | N= 203 patients | VARC-def  (72 hrs) | 19.2% | Baseline creatinine  (OR 3.8) | 30-day mortality:  2.0%, 6 months:  8.6%, 1-year:  15.5%, 2-years:  30.2% | 2-year mortality: male (HR 2.9), Stroke (HR 10), advanced AKI (HR 5.9) | no difference in AKI between IOCM and LOCM contrast media |
| Konigstein M Can J Cardiol. 2015 | Prospective observational study | N=422 consecutive patients | VARC-2-def | 15.6%  (1: 14%  2: 2%  3: 0%) | EF (OR, 0.91), PVD (OR, 2.8), and major bleeding (OR, 3.1) | 30 day mortality: 3% / 1-year: 12% | 30-day and long-term mortality: Major bleeding (HR: 6.7) / (HR: 3.3)  AKI no risk factor | no association of HT,  DM, previous MI, CHF, contrast media volume, and PRBC with AKI |
| de Brito FS Jr. Catheter Cardiovasc Interv. 2015 | multicentre register | N=418 patients | VARC-2-def | 20% | - | All-cause mortality day 30 and 1 year 9.1 and 21.5% | overall mortality: AKI (HR: 3.1), COPD (HR: 3.5), stroke (HR: 2.7), moderate/ severe para-valvular regurgitation (HR: 2.8) | 01/08 – 01/13 in 18 centers; Separation by type of mortality: early (≤30 days) mortality, late (>30 days) mortality  use of TEE - protective factor against overall (HR: 0.57) and late (HR: 0.47) mortality |
| Muñoz-García AJ  J Cardiol 2015 | retrospective study | N=357 patients | VARC-def  (72 hrs) | 15.8% | Prior stroke (OR 2.4), aortic regurgitation (OR 4.6) | 30 day mortality: 3.4%:  13.8% in AKI vs. 1.3% no AKI | AKI (HR: 2.2), stroke (HR:5.6), CHD (HR: 2.5) and Karnofsky Index (HR 0.98) | 04/08 – 12/13 |
| Barbanti M EuroIntervention. 2014 | multicentre register | N=1,157 patients | VARC-def  (72 hrs) | 20.0%  (1: 15.4%  2: 2.7%  3: 1.9%) | Female (OR: 1.4), baseline renal insufficiency (OR: 11.02), general anaesthesia (OR: 1.4) and ≥3 PRBC within 72 hrs (OR: 1.7) | - | 3-year all-cause mortality: AKI (HR: 2.1)  3-year cardiovascular mortality: AKI (HR: 2.3) | - |
| **Reference** | **Studie Design** | **Patients** | **AKI Definition** | **Incidence**  **of AKI** | **Independent predictors of AKI** | **prognostic value** | **Independent predictors of postoperative mortality** | **Further results** |
| Carrabba N Am J Cardiol. 2013 | Prospective observational study | N= 68 patients | VARC-def  (72 hrs) | 14.5% | - | - | 1-year mortality: AKI (HR 4.8) not peak cTnI | No baseline differences AKI vs no-AKI |
| Saia F Int J Cardiol. 2013 | Prospective single-center registry | N=102 consecutive patients | VARC-def  (72 hrs) | 41.7% (1: 32.4% 2: 4.9% 3: 3.9%) | Trans-apical access  HR between 4.6 and 5.2 (based on model) | All-cause mortality day 30: 4.9% | 1-year mortality: baseline eGFR <30 mL/min (HR 5.7) and AKI-3 (HR 8.6) | CKD at baseline: 87.3% different access routes (AKI: 66.7% in trans-apical, 30.3% in trans-femoral) |
| Khawaja MZ EuroIntervention. 2012 | retrospective study | N=248 patients | VARC-def  (72 hrs) | 35.9% | DM, PVD and CKD | Mortality at 30 days (13.5% vs. 3.8%) and 1-year (31.5% vs. 15.0%) | - | - |
| Kong WY Nephrology (Carlton). 2012 | retrospective study | N= 52 patients | RIFLE | 28.8%;  6% RRT | peri-procedural blood transfusion (OR: 2.4), trans-apical approach (OR: 9.3) and HT (OR: 6.4) | Cumulative mortality for AKI+ vs AKI- 26.7%, 2.7%  LOS doubled | - | improvement in eGFR: AKI- vs. AKI+ patients (70.4 vs 46.9 at 6 months and 73.7 vs 53.0 at 12 months) |
| Elhmidi Y Am Heart J. 2011 | Prospective observational study | N= 234 consecutive patients | RIFLE | 19.6%; 10.3% RRT | Preoperative creatinine (OR 3.7) |  | No multivariate analysis (unadjusted in-hospital mortality rate: 15.2% no AKI vs 7.7% with AKI | amount of contrast media was not associated with AKI |
| Nuis RJ  Catheter Cardiovasc Interv. 2011 | Prospective observational study | N= 126 consecutive patients | VARC-def  (72 hrs) | 19%  RRT 2% | Previous MI (OR: 5.7); peri-procedural RBC (OR: 1.29); post-TAVI leucocyte count (OR: 1.1); logistic EuroSCORE (OR: 1.1) | prognostic implications of peri-procedural RBC on 30 day and cumulative late mortality | AKI (OR: 5.5) and post-procedural leucocyte count (OR: 1.2) were independent predictors of 30-day mortality while AKI (HR: 2.8) was the only independent predictor of late mortality. | 5 patients on chronic HD and 3 intra-procedural deaths  30-day mortality for AKI Patients 23% and cumulative late mortality (median: 13 months) 55% |
| **Reference** | **Studie Design** | **Patients** | **AKI Definition** | **Incidence**  **of AKI** | **Independent predictors of AKI** | **prognostic value** | **Independent predictors of postoperative mortality** | **Further results** |
| Bagur R Eur Heart J 2010 | Prospective observational study | N=213 Patients with CNI  TAVI vs. SAVR | reduction of >25% in eGFR within 48 h OR need for HD during index hospitalization | Overall:  11.7%/ 1.4% | hypertension (OR: 4.7); COPD (OR: 2.6); peri-operative blood transfusion (OR: 3.5) | 9.8% died during index hospitalization | EuroSCORE (OR: 1.03) and occurrence of AKI (OR: 4.1) were identified as | Incidence of AKI after TAVI 9.2% /2.5% compared with SAVR 25.9% / 8.7% |
| Sinning JM JACC Cardiovasc Interv. 2010 | Prospective observational study | N=77 patients | AKIN | 26% | Univariate: eGFR, Peri-prosthetic regurgitation, SIRS, Leukocyte count  No Multivariate analysis | All-cause mortality 30 days and 1 year: 10%, 26%  Survival rate according to quartiles of baseline creatinine | 30 day-mortality: EuroScore (HR 1.1), STS Score (HR 1.1), baseline creatinine (Q4) – (HR 5.9), AKI (HR 4.9)  AKI for 1 year mortality highest HR of all variables in cox model (HR 5.9) | influence of baseline renal function AKI on prognosis  Median follow-up time: 283 days |

*Valve academic research consortium (VARC)-2 [Leon 2011; Kappetein 2013]; OR, odds ratio; LOS, length of stay; AKI, acute kidney injury; AKR, recovery from acute kidney injury; pAKI, persistent AKI; TAVI, transcatheter-aortic-valve-implantation; CKD, chronic kidney disease; GFR, glomerular filtration rate; DM, diabetes mellitus; AF, atrial fibrillation; STS Score, Score of the Society of Thoracic Surgeons; HR, hazard ration; KDIGO, Kidney Disease Improving Global Outcome Initiative [Kidney Int]; RIFLE, Risk, Injury, Failure, Loss of kidney function and End-stage kidney disease classification [Bellomo 2004]; AKIN, Acute kidney injury Network [Mehta 2007]; PCI, percutaneous coronary intervention; EuroScore [Nashef 1999]; IABP, intra-aortic balloon pump; BMI, body mass index; MI, myocardial infarction; HT, hypertension; IOCM / LOCM, iso-osmolar (IOCM) and low-osmolar contrast media (LOCM); EF, ejection fraction; PVD, peripheral vascular disease; CHF, congestive heart failure; PRBC, packed red blood cells; COPD, chronic obstruvtive pulmonary disease; TEE, transesophageal echocardiography; HD, hemodialysis.

**References to Appendix Table 1**

# Ando T, Adegbala O, Villablanca P, Akintoye E, Ashraf S, Shokr M et al. Incidence, Predictors, and In-Hospital Outcomes of Transcatheter Aortic Valve Implantation After Nonelective Admission in Comparison With Elective Admission: From the Nationwide Inpatient Sample Database. Am J Cardiol 2019;123(1):100-107. doi: 10.1016/j.amjcard.2018.09.023.

[Attard S](https://www.ncbi.nlm.nih.gov/pubmed/?term=Attard%20S%5BAuthor%5D&cauthor=true&cauthor_uid=30369403), [Buttigieg J](https://www.ncbi.nlm.nih.gov/pubmed/?term=Buttigieg%20J%5BAuthor%5D&cauthor=true&cauthor_uid=30369403), [Galea S](https://www.ncbi.nlm.nih.gov/pubmed/?term=Galea%20S%5BAuthor%5D&cauthor=true&cauthor_uid=30369403), [Mintoff M](https://www.ncbi.nlm.nih.gov/pubmed/?term=Mintoff%20M%5BAuthor%5D&cauthor=true&cauthor_uid=30369403), [Farrugia E](https://www.ncbi.nlm.nih.gov/pubmed/?term=Farrugia%20E%5BAuthor%5D&cauthor=true&cauthor_uid=30369403), [Cassar A](https://www.ncbi.nlm.nih.gov/pubmed/?term=Cassar%20A%5BAuthor%5D&cauthor=true&cauthor_uid=30369403). The incidence, predictors, and prognosis of acute kidney injury after transcatheter aortic valve implantation .[Clin Nephrol.](https://www.ncbi.nlm.nih.gov/pubmed/?term=30369403) 2018;90(6):373-379. doi: 10.5414/CN109544.

[Azarbal A](https://www.ncbi.nlm.nih.gov/pubmed/?term=Azarbal%20A%5BAuthor%5D&cauthor=true&cauthor_uid=30522749), [Malenka DJ](https://www.ncbi.nlm.nih.gov/pubmed/?term=Malenka%20DJ%5BAuthor%5D&cauthor=true&cauthor_uid=30522749), [Huang YL](https://www.ncbi.nlm.nih.gov/pubmed/?term=Huang%20YL%5BAuthor%5D&cauthor=true&cauthor_uid=30522749), [Ross CS](https://www.ncbi.nlm.nih.gov/pubmed/?term=Ross%20CS%5BAuthor%5D&cauthor=true&cauthor_uid=30522749), [Solomon RJ](https://www.ncbi.nlm.nih.gov/pubmed/?term=Solomon%20RJ%5BAuthor%5D&cauthor=true&cauthor_uid=30522749), [DeVries JT](https://www.ncbi.nlm.nih.gov/pubmed/?term=DeVries%20JT%5BAuthor%5D&cauthor=true&cauthor_uid=30522749), et al. Recovery of Kidney Dysfunction After Transcatheter Aortic Valve Implantation (from the Northern New England Cardiovascular Disease Study Group). [Am J Cardiol.](https://www.ncbi.nlm.nih.gov/pubmed/30522749) 2019;123(3):426-433. doi: 10.1016/j.amjcard.2018.10.042.

[Bagur R](https://www.ncbi.nlm.nih.gov/pubmed/?term=Bagur%20R%5BAuthor%5D&cauthor=true&cauthor_uid=20037180), [Webb JG](https://www.ncbi.nlm.nih.gov/pubmed/?term=Webb%20JG%5BAuthor%5D&cauthor=true&cauthor_uid=20037180), [Nietlispach F](https://www.ncbi.nlm.nih.gov/pubmed/?term=Nietlispach%20F%5BAuthor%5D&cauthor=true&cauthor_uid=20037180), [Dumont E](https://www.ncbi.nlm.nih.gov/pubmed/?term=Dumont%20E%5BAuthor%5D&cauthor=true&cauthor_uid=20037180), [De Larochellière R](https://www.ncbi.nlm.nih.gov/pubmed/?term=De%20Larochelli%C3%A8re%20R%5BAuthor%5D&cauthor=true&cauthor_uid=20037180), [Doyle D](https://www.ncbi.nlm.nih.gov/pubmed/?term=Doyle%20D%5BAuthor%5D&cauthor=true&cauthor_uid=20037180) et.al. Acute kidney injury following transcatheter aortic valve implantation: predictive factors, prognostic value, and comparison with surgical aortic valve replacement. [Eur Heart J.](https://www.ncbi.nlm.nih.gov/pubmed/?term=Bagur+R+Eur+Heart+J+2010) 2010;31(7):865-74.

Barbanti M, Latib A, Sgroi C, Fiorina C, De Carlo M, Bedogni F et.al. [Acute kidney injury after transcatheter aortic valve implantation with self-expanding CoreValve prosthesis: results from a large multicentre Italian research project.](https://www.ncbi.nlm.nih.gov/pubmed/24213329) EuroIntervention. 2014;10(1):133-40. doi: 10.4244/EIJV10I1A20.

Carrabba N, Valenti R, Migliorini A, Vergara R, Parodi G, Antoniucci D. [Prognostic value of myocardial injury following transcatheter aortic valve implantation.](https://www.ncbi.nlm.nih.gov/pubmed/23465097) Am J Cardiol. 2013;111(10):1475-81. doi: 10.1016/j.amjcard.2013.01.301.

[Chatani K](https://www.ncbi.nlm.nih.gov/pubmed/?term=Chatani%20K%5BAuthor%5D&cauthor=true&cauthor_uid=25801148), [Abdel-Wahab M](https://www.ncbi.nlm.nih.gov/pubmed/?term=Abdel-Wahab%20M%5BAuthor%5D&cauthor=true&cauthor_uid=25801148), [Wübken-Kleinfeld N](https://www.ncbi.nlm.nih.gov/pubmed/?term=W%C3%BCbken-Kleinfeld%20N%5BAuthor%5D&cauthor=true&cauthor_uid=25801148), [Gordian K](https://www.ncbi.nlm.nih.gov/pubmed/?term=Gordian%20K%5BAuthor%5D&cauthor=true&cauthor_uid=25801148), [Pötzing K](https://www.ncbi.nlm.nih.gov/pubmed/?term=P%C3%B6tzing%20K%5BAuthor%5D&cauthor=true&cauthor_uid=25801148), [Mostafa AE](https://www.ncbi.nlm.nih.gov/pubmed/?term=Mostafa%20AE%5BAuthor%5D&cauthor=true&cauthor_uid=25801148) et.al. Acute kidney injury after transcatheter aortic valve implantation: Impact of contrast agents, predictive factors, and prognostic importance in 203 patients with long-term follow-up. [J Cardiol.](https://www.ncbi.nlm.nih.gov/pubmed/?term=Chatani+K+J+Cardiol+2015) 2015;66(6):514-9. doi: 10.1016/j.jjcc.2015.02.007.

de Brito FS Jr, Carvalho LA, Sarmento-Leite R, Mangione JA, Lemos P, Siciliano A, et al. Brazilian TAVI Registry investigators. [Outcomes and predictors of mortality after transcatheter aortic valve implantation: results of the Brazilian registry.](https://www.ncbi.nlm.nih.gov/pubmed/25510532) Catheter Cardiovasc Interv. 2015;85(5):E153-62. doi: 10.1002/ccd.25778.

[D'Onofrio A](https://www.ncbi.nlm.nih.gov/pubmed/?term=D'Onofrio%20A%5BAuthor%5D&cauthor=true&cauthor_uid=26603025), [Facchin M](https://www.ncbi.nlm.nih.gov/pubmed/?term=Facchin%20M%5BAuthor%5D&cauthor=true&cauthor_uid=26603025), [Besola L](https://www.ncbi.nlm.nih.gov/pubmed/?term=Besola%20L%5BAuthor%5D&cauthor=true&cauthor_uid=26603025), [Manzan E](https://www.ncbi.nlm.nih.gov/pubmed/?term=Manzan%20E%5BAuthor%5D&cauthor=true&cauthor_uid=26603025), [Tessari C](https://www.ncbi.nlm.nih.gov/pubmed/?term=Tessari%20C%5BAuthor%5D&cauthor=true&cauthor_uid=26603025), [Bizzotto E](https://www.ncbi.nlm.nih.gov/pubmed/?term=Bizzotto%20E%5BAuthor%5D&cauthor=true&cauthor_uid=26603025) et.al. Intermediate Clinical and Hemodynamic Outcomes After Transcatheter Aortic Valve Implantation. [Ann Thorac Surg.](https://www.ncbi.nlm.nih.gov/pubmed/26603025) 2016;101(3):881-8; Dissicussion 888. doi: 10.1016/j.athoracsur.2015.08.032.

[Elhmidi Y](https://www.ncbi.nlm.nih.gov/pubmed/?term=Elhmidi%20Y%5BAuthor%5D&cauthor=true&cauthor_uid=21473973), [Bleiziffer S](https://www.ncbi.nlm.nih.gov/pubmed/?term=Bleiziffer%20S%5BAuthor%5D&cauthor=true&cauthor_uid=21473973), [Piazza N](https://www.ncbi.nlm.nih.gov/pubmed/?term=Piazza%20N%5BAuthor%5D&cauthor=true&cauthor_uid=21473973), [Hutter A](https://www.ncbi.nlm.nih.gov/pubmed/?term=Hutter%20A%5BAuthor%5D&cauthor=true&cauthor_uid=21473973), [Opitz A](https://www.ncbi.nlm.nih.gov/pubmed/?term=Opitz%20A%5BAuthor%5D&cauthor=true&cauthor_uid=21473973), [Hettich I](https://www.ncbi.nlm.nih.gov/pubmed/?term=Hettich%20I%5BAuthor%5D&cauthor=true&cauthor_uid=21473973) et.al. Incidence and predictors of acute kidney injury in patients undergoing transcatheter aortic valve implantation. [Am Heart J.](https://www.ncbi.nlm.nih.gov/pubmed/?term=Elhmidi+Y+Am+Heart+J.+2011) 2011;161(4):735-9. doi: 10.1016/j.ahj.2011.01.009.

Ferro CJ,  Law JP,  Doshi SN, de Belder M, Moat N , Mamas M et al.   Dialysis Following Transcatheter Aortic Valve Replacement: Risk Factors and Outcomes: An Analysis From the UK TAVI (Transcatheter Aortic Valve Implantation) Registry. JACC Cardiovasc Interv. 2017;10(20):2040-2047. doi: 10.1016/j.jcin.2017.05.020.

Giannini F, Latib A, Jabbour RJ, Ruparelia N, Aurelio A, Ancona MB et. al. [Impact of post-procedural hyperglycemia on acute kidney injury after transcatheter aortic valve implantation.](https://www.ncbi.nlm.nih.gov/pubmed/27434367) Int J Cardiol. 2016;221:892-7. doi: 10.1016/j.ijcard.2016.07.029.

Kappetein AP, Head SJ, Généreux P, Piazza N, van Mieghem NM, Blackstone EH, et al. Valve Academic Research Consortium-2. [Updated standardized endpoint definitions for transcatheter aortic valve implantation: the Valve Academic Research Consortium-2 consensus document.](https://www.ncbi.nlm.nih.gov/pubmed/23084102) J Thorac Cardiovasc Surg. 2013;145(1):6-23. doi: 10.1016/j.jtcvs.2012.09.002.

[Khawaja MZ](https://www.ncbi.nlm.nih.gov/pubmed/?term=Khawaja%20MZ%5BAuthor%5D&cauthor=true&cauthor_uid=22995082), [Thomas M](https://www.ncbi.nlm.nih.gov/pubmed/?term=Thomas%20M%5BAuthor%5D&cauthor=true&cauthor_uid=22995082), [Joshi A](https://www.ncbi.nlm.nih.gov/pubmed/?term=Joshi%20A%5BAuthor%5D&cauthor=true&cauthor_uid=22995082), [Asrress KN](https://www.ncbi.nlm.nih.gov/pubmed/?term=Asrress%20KN%5BAuthor%5D&cauthor=true&cauthor_uid=22995082), [Wilson K](https://www.ncbi.nlm.nih.gov/pubmed/?term=Wilson%20K%5BAuthor%5D&cauthor=true&cauthor_uid=22995082), [Bolter K](https://www.ncbi.nlm.nih.gov/pubmed/?term=Bolter%20K%5BAuthor%5D&cauthor=true&cauthor_uid=22995082) et.al. The effects of VARC-defined acute kidney injury after transcatheter aortic valve implantation (TAVI) using the Edwards bioprosthesis. [EuroIntervention.](https://www.ncbi.nlm.nih.gov/pubmed/?term=Khawaja+MZ+EuroIntervention.+2012) 2012;8(5):563-70. doi: 10.4244/EIJV8I5A87.

Konigstein M, Ben-Assa E, Banai S, Shacham Y, Ziv-Baran T et.al. P[eriprocedural bleeding, acute kidney injury, and long-term mortality after transcatheter aortic valve implantation.](https://www.ncbi.nlm.nih.gov/pubmed/25547551) Can J Cardiol. 2015;31(1):56-62. doi: 10.1016/j.cjca.2014.11.006.

[Kong WY](https://www.ncbi.nlm.nih.gov/pubmed/?term=Kong%20WY%5BAuthor%5D&cauthor=true&cauthor_uid=22390156), [Yong G](https://www.ncbi.nlm.nih.gov/pubmed/?term=Yong%20G%5BAuthor%5D&cauthor=true&cauthor_uid=22390156), [Irish A](https://www.ncbi.nlm.nih.gov/pubmed/?term=Irish%20A%5BAuthor%5D&cauthor=true&cauthor_uid=22390156). Incidence, risk factors and prognosis of acute kidney injury after transcatheter aortic valve implantation. [Nephrology (Carlton).](https://www.ncbi.nlm.nih.gov/pubmed/?term=Kong+WY+Nephrology+(Carlton).+2012" \o "Nephrology (Carlton, Vic.).) 2012;17(5):445-51. doi: 10.1111/j.1440-1797.2012.01593.x.

Kumar N, Khera R, Fonarow GC, Bhatt DL. Comparison of Outcomes of Transfemoral Versus Transapical Approach for Transcatheter Aortic Valve Implantation. Am J Cardiol. 2018;122(9):1520-1526. doi: 10.1016/j.amjcard.2018.07.025.

Leon MB, Piazza N, Nikolsky E, et al. Standardized endpoint definitions for transcatheter aortic valve implantation clinical trials: a consensus report from the Valve Academic Research Consortium. Eur Heart J 2011;32:105-17.

[Muñoz-García AJ](https://www.ncbi.nlm.nih.gov/pubmed/?term=Mu%C3%B1oz-Garc%C3%ADa%20AJ%5BAuthor%5D&cauthor=true&cauthor_uid=25454207), [Muñoz-García E](https://www.ncbi.nlm.nih.gov/pubmed/?term=Mu%C3%B1oz-Garc%C3%ADa%20E%5BAuthor%5D&cauthor=true&cauthor_uid=25454207), [Jiménez-Navarro MF](https://www.ncbi.nlm.nih.gov/pubmed/?term=Jim%C3%A9nez-Navarro%20MF%5BAuthor%5D&cauthor=true&cauthor_uid=25454207), [Domínguez-Franco AJ](https://www.ncbi.nlm.nih.gov/pubmed/?term=Dom%C3%ADnguez-Franco%20AJ%5BAuthor%5D&cauthor=true&cauthor_uid=25454207), [Alonso-Briales JH](https://www.ncbi.nlm.nih.gov/pubmed/?term=Alonso-Briales%20JH%5BAuthor%5D&cauthor=true&cauthor_uid=25454207), [Hernández-García JM](https://www.ncbi.nlm.nih.gov/pubmed/?term=Hern%C3%A1ndez-Garc%C3%ADa%20JM%5BAuthor%5D&cauthor=true&cauthor_uid=25454207) et. al. Clinical impact of acute kidney injury on short- and long-term outcomes after transcatheter aortic valve implantation with the CoreValve prosthesis. [J Cardiol.](https://www.ncbi.nlm.nih.gov/pubmed/?term=Mu%C3%B1oz-Garc%C3%ADa+AJ+J+Cardiol+2015) 2015;66(1):46-9. doi: 10.1016/j.jjcc.2014.09.009.

Nashef SA, Roques F, Michel P, Gauducheau E, Lemeshow S, Salamon R. [European system for cardiac operative risk evaluation (EuroSCORE).](https://www.ncbi.nlm.nih.gov/pubmed/10456395) Eur J Cardiothorac Surg. 1999;16(1):9-13.

[Nijenhuis VJ](https://www.ncbi.nlm.nih.gov/pubmed/?term=Nijenhuis%20VJ%5BAuthor%5D&cauthor=true&cauthor_uid=29525062), [Peper J](https://www.ncbi.nlm.nih.gov/pubmed/?term=Peper%20J%5BAuthor%5D&cauthor=true&cauthor_uid=29525062), [Vorselaars VMM](https://www.ncbi.nlm.nih.gov/pubmed/?term=Vorselaars%20VMM%5BAuthor%5D&cauthor=true&cauthor_uid=29525062), [Swaans MJ](https://www.ncbi.nlm.nih.gov/pubmed/?term=Swaans%20MJ%5BAuthor%5D&cauthor=true&cauthor_uid=29525062), [De Kroon T](https://www.ncbi.nlm.nih.gov/pubmed/?term=De%20Kroon%20T%5BAuthor%5D&cauthor=true&cauthor_uid=29525062), [Van der Heyden JAS](https://www.ncbi.nlm.nih.gov/pubmed/?term=Van%20der%20Heyden%20JAS%5BAuthor%5D&cauthor=true&cauthor_uid=29525062) et. al. Prognostic Value of Improved Kidney Function After Transcatheter Aortic Valve Implantation for Aortic Stenosis. [Am J Cardiol.](https://www.ncbi.nlm.nih.gov/pubmed/?term=Nijenhuis+VJ+Am+J+Cardiol.+2018) 2018;121(10):1239-1245. doi: 10.1016/j.amjcard.2018.01.049.

[Nuis RJ](https://www.ncbi.nlm.nih.gov/pubmed/?term=Nuis%20RJ%5BAuthor%5D&cauthor=true&cauthor_uid=21061244), [Van Mieghem NM](https://www.ncbi.nlm.nih.gov/pubmed/?term=Van%20Mieghem%20NM%5BAuthor%5D&cauthor=true&cauthor_uid=21061244), [Tzikas A](https://www.ncbi.nlm.nih.gov/pubmed/?term=Tzikas%20A%5BAuthor%5D&cauthor=true&cauthor_uid=21061244), [Piazza N](https://www.ncbi.nlm.nih.gov/pubmed/?term=Piazza%20N%5BAuthor%5D&cauthor=true&cauthor_uid=21061244), [Otten AM](https://www.ncbi.nlm.nih.gov/pubmed/?term=Otten%20AM%5BAuthor%5D&cauthor=true&cauthor_uid=21061244), [Cheng J](https://www.ncbi.nlm.nih.gov/pubmed/?term=Cheng%20J%5BAuthor%5D&cauthor=true&cauthor_uid=21061244) et.al. Frequency, determinants, and prognostic effects of acute kidney injury and red blood cell transfusion in patients undergoing transcatheter aortic valve implantation. [Catheter Cardiovasc Interv.](https://www.ncbi.nlm.nih.gov/pubmed/21061244) 2011;77(6):881-9. doi: 10.1002/ccd.22874.

[Nunes Filho ACB](https://www.ncbi.nlm.nih.gov/pubmed/?term=Nunes%20Filho%20ACB%5BAuthor%5D&cauthor=true&cauthor_uid=29358043), [Katz M](https://www.ncbi.nlm.nih.gov/pubmed/?term=Katz%20M%5BAuthor%5D&cauthor=true&cauthor_uid=29358043), [Campos CM](https://www.ncbi.nlm.nih.gov/pubmed/?term=Campos%20CM%5BAuthor%5D&cauthor=true&cauthor_uid=29358043), [Carvalho LA](https://www.ncbi.nlm.nih.gov/pubmed/?term=Carvalho%20LA%5BAuthor%5D&cauthor=true&cauthor_uid=29358043), [Siqueira DA](https://www.ncbi.nlm.nih.gov/pubmed/?term=Siqueira%20DA%5BAuthor%5D&cauthor=true&cauthor_uid=29358043), [Tumelero RT](https://www.ncbi.nlm.nih.gov/pubmed/?term=Tumelero%20RT%5BAuthor%5D&cauthor=true&cauthor_uid=29358043) et al. Impact of Acute Kidney Injury on Short- and Long-term Outcomes After Transcatheter Aortic Valve Implantation. [Rev Esp Cardiol (Engl Ed).](https://www.ncbi.nlm.nih.gov/pubmed/?term=Nunes+Filho+ACB+Rev+Esp+Cardiol+(Engl+Ed).+2019" \o "Revista espanola de cardiologia (English ed.).) 2019;72(1):21-29. doi: 10.1016/j.rec.2017.11.024.

Saia F, Ciuca C, Taglieri N, Marrozzini C, Savini C, Bordoni B, et al. [Acute kidney injury following transcatheter aortic valve implantation: incidence, predictors and clinical outcome.](https://www.ncbi.nlm.nih.gov/pubmed/23164594) Int J Cardiol. 2013;168(2):1034-40. doi: 10.1016/j.ijcard.2012.10.029.

[Sinning JM](https://www.ncbi.nlm.nih.gov/pubmed/?term=Sinning%20JM%5BAuthor%5D&cauthor=true&cauthor_uid=21087750), [Ghanem A](https://www.ncbi.nlm.nih.gov/pubmed/?term=Ghanem%20A%5BAuthor%5D&cauthor=true&cauthor_uid=21087750), [Steinhäuser H](https://www.ncbi.nlm.nih.gov/pubmed/?term=Steinh%C3%A4user%20H%5BAuthor%5D&cauthor=true&cauthor_uid=21087750), [Adenauer V](https://www.ncbi.nlm.nih.gov/pubmed/?term=Adenauer%20V%5BAuthor%5D&cauthor=true&cauthor_uid=21087750), [Hammerstingl C](https://www.ncbi.nlm.nih.gov/pubmed/?term=Hammerstingl%20C%5BAuthor%5D&cauthor=true&cauthor_uid=21087750), [Nickenig G](https://www.ncbi.nlm.nih.gov/pubmed/?term=Nickenig%20G%5BAuthor%5D&cauthor=true&cauthor_uid=21087750) et.al. Renal function as predictor of mortality in patients after percutaneous transcatheter aortic valve implantation. [JACC Cardiovasc Interv.](https://www.ncbi.nlm.nih.gov/pubmed/?term=Sinning+JM+JACC+Cardiovasc+Interv.+2010) 2010;3(11):1141-9. doi: 10.1016/j.jcin.2010.09.009.

[Thongprayoon C](https://www.ncbi.nlm.nih.gov/pubmed/?term=Thongprayoon%20C%5BAuthor%5D&cauthor=true&cauthor_uid=28833503), [Cheungpasitporn W](https://www.ncbi.nlm.nih.gov/pubmed/?term=Cheungpasitporn%20W%5BAuthor%5D&cauthor=true&cauthor_uid=28833503), [Mao MA](https://www.ncbi.nlm.nih.gov/pubmed/?term=Mao%20MA%5BAuthor%5D&cauthor=true&cauthor_uid=28833503), [Srivali N](https://www.ncbi.nlm.nih.gov/pubmed/?term=Srivali%20N%5BAuthor%5D&cauthor=true&cauthor_uid=28833503), [Kittanamongkolchai W](https://www.ncbi.nlm.nih.gov/pubmed/?term=Kittanamongkolchai%20W%5BAuthor%5D&cauthor=true&cauthor_uid=28833503), [Harrison AM](https://www.ncbi.nlm.nih.gov/pubmed/?term=Harrison%20AM%5BAuthor%5D&cauthor=true&cauthor_uid=28833503) et.al. Persistent acute kidney injury following transcatheter aortic valve replacement. [J Card Surg.](https://www.ncbi.nlm.nih.gov/pubmed/?term=Thongprayoon+C+J+Card+Surg.+2017) 2017;32(9):550-555. doi: 10.1111/jocs.13200.

[Vavilis G](https://www.ncbi.nlm.nih.gov/pubmed/?term=Vavilis%20G%5BAuthor%5D&cauthor=true&cauthor_uid=28761674), [Evans M](https://www.ncbi.nlm.nih.gov/pubmed/?term=Evans%20M%5BAuthor%5D&cauthor=true&cauthor_uid=28761674), [Jernberg T](https://www.ncbi.nlm.nih.gov/pubmed/?term=Jernberg%20T%5BAuthor%5D&cauthor=true&cauthor_uid=28761674), [Rück A](https://www.ncbi.nlm.nih.gov/pubmed/?term=R%C3%BCck%20A%5BAuthor%5D&cauthor=true&cauthor_uid=28761674), [Szummer K](https://www.ncbi.nlm.nih.gov/pubmed/?term=Szummer%20K%5BAuthor%5D&cauthor=true&cauthor_uid=28761674). Risk factors for worsening renal function and their association with long-term mortality following transcatheter aortic valve implantation: data from the SWEDEHEART registry. [Open Heart.](https://www.ncbi.nlm.nih.gov/pubmed/28761674) 2017;4(2):e000554. doi: 10.1136/openhrt-2016-000554. eCollection 2017.
